# Acoustic Change Complex Evoked by Horizontal Sound Location Change in Young Adults With Normal Hearing

**DOI:** 10.3389/fnins.2022.908989

**Published:** 2022-06-06

**Authors:** Zhi-Tong Fan, Zi-Hui Zhao, Mridula Sharma, Joaquin T. Valderrama, Qian-Jie Fu, Jia-Xing Liu, Xin Fu, Huan Li, Xue-Lei Zhao, Xin-Yu Guo, Luo-Yi Fu, Ning-Yu Wang, Juan Zhang

**Affiliations:** ^1^Department of Otolaryngology Head and Neck Surgery, Beijing Chaoyang Hospital, Capital Medical University, Beijing, China; ^2^Department of Linguistics, Faculty of Human Sciences, Macquarie University, Sydney, NSW, Australia; ^3^National Acoustic Laboratories, Sydney, NSW, Australia; ^4^Department of Head and Neck Surgery, David Geffen School of Medicine, University of California, Los Angeles, Los Angeles, CA, United States

**Keywords:** acoustic change complex, sound localization, minimum audible angle, sound localization discrimination, auditory evoked potential

## Abstract

Acoustic change complex (ACC) is a cortical auditory-evoked potential induced by a change of continuous sound stimulation. This study aimed to explore: (1) whether the change of horizontal sound location can elicit ACC; (2) the relationship between the change of sound location and the amplitude or latency of ACC; (3) the relationship between the behavioral measure of localization, minimum audible angle (MAA), and ACC. A total of 36 normal-hearing adults participated in this study. A 180° horizontal arc-shaped bracket with a 1.2 m radius was set in a sound field where participants sat at the center. MAA was measured in a two-alternative forced-choice setting. The objective electroencephalography recording of ACC was conducted with the location changed at four sets of positions, ±45°, ±15°, ±5°, and ±2°. The test stimulus was a 125–6,000 Hz broadband noise of 1 s at 60 ± 2 dB SPL with a 2 s interval. The N1′–P2′ amplitudes, N1′ latencies, and P2′ latencies of ACC under four positions were evaluated. The influence of electrode sites and the direction of sound position change on ACC waveform was analyzed with analysis of variance. Results suggested that (1) ACC can be elicited successfully by changing the horizontal sound location position. The elicitation rate of ACC increased with the increase of location change. (2) N1′–P2′ amplitude increased and N1′ and P2′ latencies decreased as the change of sound location increased. The effects of test angles on N1′–P2′ amplitude [*F*(1.91,238.1) = 97.172, *p* < 0.001], N1′ latency [*F*(1.78,221.90) = 96.96, *p* < 0.001], and P2′ latency [*F*(1.87,233.11) = 79.97, *p* < 0.001] showed a statistical significance. (3) The direction of sound location change had no significant effect on any of the ACC peak amplitudes or latencies. (4) Sound location discrimination threshold by the ACC test (97.0% elicitation rate at ±5°) was higher than MAA threshold (2.08 ± 0.5°). The current study results show that though the ACC thresholds are higher than the behavioral thresholds on MAA task, ACC can be used as an objective method to evaluate sound localization ability. This article discusses the implications of this research for clinical practice and evaluation of localization skills, especially for children.

## Introduction

Sound localization and hearing acuity together constitute a complete auditory function. Sound localization is an important ability to determine the source, distance, and speed of sounds. It is difficult for cochlear implant and/or hearing aid users to be alert to environmental sounds or to perform well in a noisy environment, which is closely related to their compromised sound localization ability. Since sound localization ability can indicate life quality and the effectiveness of auxiliary devices, more researchers have paid attention to sound localization to explore its mechanism and clinical evaluations. The present study aimed to investigate the efficacy of an objective assessment as an index of localization.

Two main categories of subjective sound localization measurements are commonly employed in research. One is the source azimuth discrimination method, which measures the minimum audible angle (MAA) at symmetrical sound positions. The other is the source azimuth identification method, which evaluates the ability to localize sounds in terms of localization accuracy and reaction time. Concerning MAA, its thresholds can be measured using a two-alternative forced-choice task where participants point out the sound (Grieco-Calub et al., 2008) or turn their heads toward the sound (Muir et al., 1989), making it easier for children to perform when combining visual reinforcement or play audiometry. As for source localization, researchers usually adopt a closed-set response option where loudspeakers are positioned along an arc in the frontal hemifield and the separation angle between loudspeakers varies across studies, being as small as 10° and as large as 45° (Litovsky and Godar, 2010; Zheng et al., 2015; Cullington et al., 2017).

Although subjective sound localization discrimination and identification measurements have been widely used, they do not meet research and clinic needs in full. The main drawback of these behavioral measures is that they both require participants’ active involvement, making it difficult to assess certain segments of the population who cannot cooperate, such as young children or people with communication disabilities. Moreover, behavioral tests are dependent on extraneous factors such as age, intelligence, attention, and motivation. In contrast, objective measures based on electrophysiology have a strong potential in the evaluation of sound localization ability, as they require minimum interest, participation, and/or attention.

The acoustic change complex (ACC) is a cortical auditory response and was first reported by Ostroff et al. (1998). In adults, ACC is observed as three clear peaks P1′–N1′–P2′ complex with latencies around 50, 100, and 180–200 ms, respectively, following the change onset. ACC is elicited by the acoustic–physical property change of a continuous stimulus where the change can occur in (1) frequency, intensity, period, or interval of a non-speech stimulus; (2) vowel’s second formant (e.g., /u-i/), consonant–vowel syllable (e.g., /sei/), or continuous syllable (e.g., /dada/) of a speech stimulus; (3) frequency modulation or spectrum (Ostroff et al., 1998; Martin and Boothroyd, 1999, 2000; Small and Werker, 2012; Han and Dimitrijevic, 2015; Kirby and Brown, 2015; Kalaiah, 2018); and (4) gap detection (He et al., 2012). Being an objective and passive measurement, ACC has a broad clinical prospect. In auditory development, researchers have used ACC to assess infants’ speech recognition abilities and their speech perception differences under different stimuli (Small and Werker, 2012; Chen and Small, 2015; McCarthy et al., 2019). ACC can provide some insights into assessing the speech perception ability of hearing aid users and cochlear implant users (Friesen and Tremblay, 2006; Tremblay et al., 2006; Martin, 2007; Miller and Zhang, 2014; Liang et al., 2018; Shetty and Puttabasappa, 2020), and in evaluating the efficacy of assistive listening devices such as hearing aids (Brown et al., 2015; Kirby and Brown, 2015). It has also been used in investigations such as cochlear dead region measurement (Kang et al., 2018) and the objective assessment of tinnitus (Han et al., 2017).

Although ACC has been explored for several years, previous studies have mainly focused on acoustic–physical parameters such as a frequency change, and little is known about ACC’s application in sound localization. Theoretically, the location change of an auditory stimulus can also evoke ACC but whether a sound location change elicits an ACC requires investigation. While this study was in progress, Zhang et al. (2021) reported that an ACC response was evoked by a change in the stimulus location, which encouraged further exploration of the clinical setting and applications of ACC in sound localization. It remained unclear whether and how ACC amplitude and latency changed as the sound location changed, or the relationship between objective location change induced ACC and subjective sound localization behavioral measurements. Since objective testing of sound source localization ability is an urgent clinical need, this study mainly focused on whether and how the change of horizontal sound location elicits an ACC response, and the relationship between its morphology and behavioral outcomes from an MAA test.

## Materials and Methods

### Participants

A total of 36 normal hearing participants with good general health were recruited by the Hearing Center at Beijing Chaoyang Hospital, Capital Medical University (Beijing, China), from 2019 to 2020. All participants’ hearing thresholds across the audiometric frequencies from 125 to 8,000 Hz were below 20 dB HL in both ears, measured *via* an Interacoustics AC40 audiometer. Type A results were recorded in all participants measured using an Interacoustics AT235 tympanometer. A total of 34 participants were right-handed and 2 were left-handed as measured by the Edinburgh Handedness Inventory (Oldfield, 1971). Participants were between 20 and 44 years of age (mean = 25, SD = 4.75), including 12 men and 24 women. This study was approved by the Ethics Committee of Beijing Chaoyang Hospital (approval number: 2019-349). All participants signed a consent form before participating. Three participants fell asleep during the experiment and the data were not included.

### Test Environment

**FIGURE 1:**
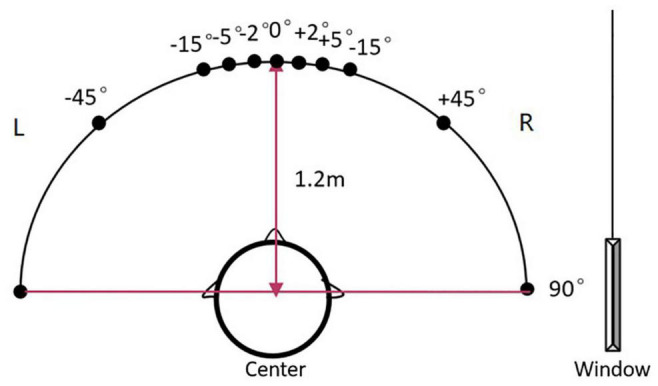
The diagram of the sound localization test. The sound localization test used a 180° horizontal arc-shaped bracket (black line) with a 1.2 m radius to place loudspeakers. The middle point was marked as 0° and four symmetrical positions (±45°, ±15°, ±5°, and ±2°) were selected as test angles. Two calibrated speakers (4 cm diameter) with the same frequency response were symmetrically fixed on the left and right sides of the bracket. Participants were asked to seat at the center of the arc facing 0°. During the test, the stimulus was played by two speakers at one of the symmetrical positions in order. For example, a stimulus was played from the speakers at –45° for the first 500 ms, then was played from the speakers at +45° for the last 500 ms and made a location change.

The localization set-up included a 180° horizontal arc-shaped bracket with a 1.2 m radius and the middle point was marked as 0° (Figure 1). Participants were seated at the center and were instructed to look forward without moving their head or body. Two speakers (Lifetrons DrumBass III BT Speaker, 4 cm diameter) with the same frequency response were symmetrically fixed on the left and right sides of the bracket, and the central position of the speakers was placed at the ear level. Speaker calibration was undertaken before each test using white noise with a ±1 dB tolerance range where a sound level meter was placed at participants’ head level. Four symmetrical positions (±45°, ±15°, ±5°, and ±2°) were selected as test angles. Speakers were placed in two symmetrical positions in each round of tests. All tests were conducted in an electrically shielded and sound-attenuated booth (2.5 m × 2.3 m, ≤30 dB A) at the Beijing Chaoyang Hospital.

### Behavioral Test

The MAA was used to measure the minimum angle that a participant could identify at 0° azimuth. Due to the speaker size in this study, the minimum MAA test angle was ±2°. The test stimulus was 125–6,000 Hz broadband noise of 1 s at 60 ± 2 dB SPL. It was generated using Adobe Audition CC software (version 6.0) at a sampling rate of 44,100 Hz and 16-bit depth. The test included a practice trial at the largest angle (±45°) to ensure participants fully understood the test. A two-alternative forced-choice task was used in MAA testing and participants were required to locate the sound source. The stimulus was presented 10 times at each angle, with a random presenting location each time, and 5 times on each side. If the accuracy rate was 80% and above, the participant was considered to be able to locate two different sounds at that angle successfully; therefore, a smaller test angle was selected to continue the test. If the participant could not locate the sound, a wider test angle was used to continue the test. The test stopped when the accuracy rate reached 80%, and that test angle was noted as the participant’s minimum audible angle.

### Acoustic Change Complex Test

#### Acoustic Change Complex Test Method

The test stimulus used in ACC was the same broadband noise as used in MAA test (125–6,000 Hz, 1 s duration, and 60 ± 2 dB SPL intensity). The first 500 ms section of the stimulus was played from the left speaker and the last 500 ms section from the right, and *vice versa*, followed by a mute interval of 2,000 ms, thus making the presentation period 3,000 ms. The beginning and the end of the stimulus had a 50 ms fade-in and a 50 ms fade-out process to avoid sudden sound appearance and disappearance. There was a programmed 1 ms overlap when the sound location change occurred, resulting in a smooth sound transition to ensure ACC was elicited by the location change instead of a “click” noise.

During the test, participants were instructed to orient their face to the front speaker (0° azimuth) and to remain still and awake to ensure clear EEG signals were recorded. Participants were also required to switch their electronic devices to airplane mode. Researchers maintained a vigil on the participants from a window and gave participants all the breaks they required. Three participants fell asleep during the test, and their data were not included in this analysis.

#### Acoustic Change Complex Test Conditions

The ACC test presented stimulus from four symmetrical locations (±45°, ±15°, ±5°, and ±2°), providing a rightward or leftward change in sound location. There were 8 test conditions (4 test angles × 2 directions of sound location change), expressed as [−45°, +45°], [+45°, −45°], [−15°, +15°], [+15°, −15°], [−5°, +5°], [+5°, −5°], [−2°, +2°], and [+2°, −2°] (Table 1). For example, [−45°, +45°] represented the stimulus was presented from −45° in the first 500 ms and from +45° in the last 500 ms with a total 90° sound location change. The stimulus playback control was produced by E-prime Software 2.0, American Psychological Software Tools. There were eight rounds of the test, and each condition’s test order was random. Each presentation duration was 3 s. Considering the 200 times repetitions, each round of the test duration was approximately 10 min (200 times × 3 s = 10 min), and the total test duration was 80 min (10 min × 8 rounds = 80 min). Participants had a 10–20 min break every half an hour to prevent fatigue from affecting results.

**TABLE 1:** Eight test conditions.

Condition	1	2	3	4	5	6	7	8
Location 1	−45°	+45°	−15°	+15°	−5°	+5°	−2°	+2°
Location 2	+45°	−45°	+15°	−15°	+5°	−5°	+2°	−2°

#### EEG Acquisition Settings

Considering that frontal or central midline electrode sites tend to have maximal cortical auditory-evoked potential (CAEP) amplitudes (Itoh et al., 2015; Peter et al., 2019) and mastoid sites pick up small cortical activity from that area, EEG responses were recorded by Ag/AgCl electrodes using six fronto-central (Cz, Fz, F3, F4, C3, C4) active electrodes referenced to the right mastoid (M2). The ground electrode was placed on the forehead. Each electrode impedance was maintained below 5 kΩ. The EEG SynAmps2 amplifier (Neuroscan) and Curry 7 software (version 7.0.9) were used for the acquisition of the EEG signals, using a sampling rate of 1 kHz.

#### Acoustic Change Complex Waveform Analysis and Interpretation

The Curry 7 software was used for data analysis. Constant baseline correction was performed with a [−200 to 0] ms time window. Data were filtered offline using a 1–30 Hz bandpass filter to reduce the effect of non-relevant myoelectrical signals and the 50-Hz AC component. Artifact reduction was achieved by identifying and removing noisy EEG epochs (i.e., those with values outside the ±100 μV range) from averaging, and mechanical waves were removed by template matching when applicable. The analysis time window was 2,000 ms to avoid any possible overlapping with the following epoch. The potentials were processed and averaged after 200 repetitions. Amplitude was defined in terms of the peak-to-baseline distance, and latency was measured as the time difference between 0 ms and the peak. The first set of components within the [0–300] ms was CAEP to sound onset, whereas ACC occurred at 500–800 ms as a result of the sound location change at 500 ms. ACC’s P1′, N1′, and P2′ components would appear at around 550, 600, and [680–800] ms, respectively. Two researchers interpreted waveforms independently to ensure reliability in peak evaluation. When the two researchers agreed with peak identification, the result was accepted. When they disagreed, a third opinion was considered. In addition to the subjective (visual) identification of the P1–N1–P2 components by expert evaluators, the determination of a neurophysiological response in the recorded signal was also conducted *via* an objective method based on the correlation coefficient (Pearson-*r*) between the responses obtained when the stimulus transitioned from the left to the right speaker, and from the right to the left speaker. This objective method provides an index of response replication – Pearson-*r* values greater than 0.7 being associated with the presence of neurophysiological response (Weber and Fletcher, 1980; Elberling and Don, 2007). The analysis of the correlation coefficient was conducted in the onset-CAEP and the ACC obtained with the Cz electrode in the ±45° test condition, in the time range [100–300] ms for the onset-CAEP and [600–800] ms for the ACC.

Since P1′ waveform was less noticeable compared to N1′ and P2′, the data analysis focused on the N1′–P2′ amplitude (i.e., amplitude P2′ – amplitude N1′), N1′ latency, and P2′ latency. Data recorded from Cz and Fz were used for analysis as they were more easily recognized than other recording sites.

### Statistical Analysis

The SPSS software (version 23.0) was performed for data analysis, using repeated measures analysis of variance to analyze N1–P2 amplitude, N1 latency, and P2 latency under different conditions, different test angles as the within-subjects variables, the electrode position (Cz and Fz), and the direction of sound movement as within-subjects factors. If the data were not satisfactory with the spherical distribution hypothesis, the Greenhouse Geisel method correction was applied. Statistical significance was achieved with *p* < 0.05.

## Results

### Subjective and Objective Determination of Neurophysiological Response

**FIGURE 2:**
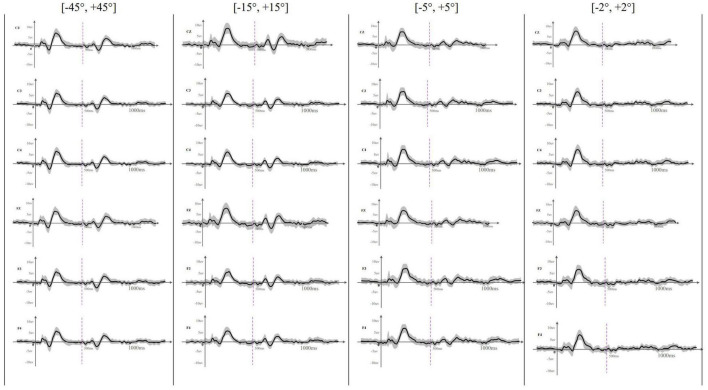
The averaged (*n* = 33) waveform at all recording electrodes elicited by rightward location change in different test angles. This figure showed the averaged waveform obtained at Cz, C3, C4, Fz, F3, and F4 across different location change angles. The sound location change happened at 500 ms and was indicated by a purple dotted line. The SD of each averaged waveform was plotted in a gray area. Onset-CAEP appeared within 50–250 ms and ACC appeared within 600–800 ms except that ACC waveform was difficult to judge at [–2°, +2°].

Figure 2 presents the grand-average onset-CAEP and ACC signals obtained across participants at different electrode sites and angle-difference conditions. This figure visually shows that the morphology of both the onset-CAEP and the ACC is consistent with the expected waveform and that the P1–N1–P2 components can be visually identified. Consistent with the subjective (visual) identification of the responses, the high values of the correlation coefficient analysis conducted in the onset-CAEP and ACC signals in the ±45° test condition at the Cz electrode (Pearson-*r* of onset-CAEP: mean = 0.89, STD = 0.12; median = 0.93; Pearson-*r* of ACC: mean = 0.80, STD = 0.17; median = 0.84) objectively demonstrated the presence of neurophysiological response.

### Onset-Cortical Auditory-Evoked Potential

EEG results showed that the onset-CAEPs P1–N1–P2 components could be observed in all the participants. The response waveform had a consistent morphology and similar amplitudes and latencies across different localization conditions in all the electrodes (Figure 2). Results also showed that the starting side of the stimulus presentation (left first or right first) had no statistically significant influence on the N1–P2 amplitude [*F*(1,129) = 2.11, *p* = 0.15 > 0.05, η = 0.016], N1 latency [*F*(1,129) = 0.075, *p* = 0.78 > 0.05, η = 0.001], and P2 latency [*F*(1,129) = 1.14, *p* = 0.29 > 0.05, η = 0.009], so onset-CAEP at different angles were analyzed together. Table 2 shows that the N1–P2 amplitude of onset-CAEP is statistically different between each test angle, while there is no significant difference in the latency of N1 or P2.

**TABLE 2:** Comparisons of onset-CAEP between different test angles.

	N1–P2 amplitude (μV)			N1 latency (ms)			P2 latency (ms)		
Angle (°)	MD	SE	*p*	MD	SE	*p*	MD	SE	*p*
±5 vs. ±15	−0.44	0.16	0.006**	0.59	1.54	0.70	−0.49	1.46	0.74
±15 vs. ±45	−0.82	0.18	<0.001***	−0.91	1.29	0.48	−16.96	15.26	0.27
±5 vs. ±45	−1.26	0.19	<0.001***	−0.32	1.38	0.81	−17.45	15.32	0.26

*The table shows the comparison results of onset-CAEP N1–P2 amplitude, N1 latency, and P2 latency under different test angles (±5°, ±15°, and ±45°) via the analysis of variance. It can be seen that the N1–P2 amplitude increased with the increase of the test angle. There was a significant statistical difference (p < 0.001) between ±15° and ±45°, and ±5°, and ±45°, and also a statistical difference (0.001 ≤ p < 0.01) between ±5° and ±15°. Meanwhile, N1 latency and P2 latency showed no statistical differences (p < 0.001) between different test angles.*

MD, mean difference; SE, standard error; **, 0.001 ≤ p < 0.01; ***, p < 0.001.

### Acoustic Change Complex Can Be Evoked by Sound Location Changes at Different Angles

**FIGURE 3:**
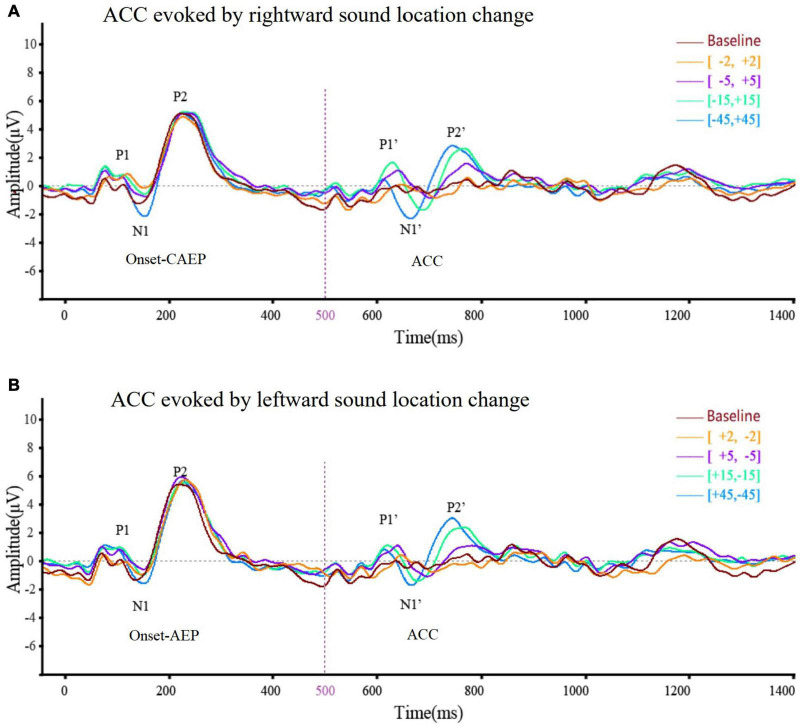
The averaged (*n* = 33) ACC waveform at Cz elicited by **(A)** rightward location change and **(B)** leftward location change across different test angles. This figure showed the overlying waveform of EEG (*n* = 33) at electrode Cz while the sound location change was rightward **(A)** and leftward **(B)**. The ordinate represented the amplitude of the waveform, and the positive value was upward. The abscissa represented one sound playing cycle. The sound started playing at 0 ms, changed its position at 500 ms, and ended at 1,000 ms. The onset-CAEP appeared within 50–250 ms. A similar waveform, which was ACC, appeared within 600–800 ms (the sound change happened at 500 ms). Clear waveforms can be observed under the conditions of [–45°, +45°] (blue line), [–15°, +15°] (green line), [–5°, +5°] (purple line), and [–2°, +2°] (yellow line). Baseline waveform was obtained by presenting the same stimulus without any changes to 10 of 33 participants at –45°. The CAEP waveforms elicited under different conditions almost coincided. After overlapping ACC waveforms under different conditions, it can be seen that the latency of N1′ and P2′ tends to shorten and the N1′–P2′ amplitude tends to increase with the increase of test angle regardless of the location change direction.

Acoustic change complex evoked by a sound location change was observed under all eight test conditions in all the participants. Table 3 presents the amplitude and latency of the N1′ and P2′ components. There were similar ACC waveforms across all electrodes at each rightward location change (Figure 2). As shown in Figure 3, there was a P1′–N1′–P2′ waveform in 500–800 ms, of which the waveforms at [−45°, +45°], [−15°, +15°], and [−5°, +5°] were significant, while the waveform at [−2°, +2°] was less obvious. A baseline waveform was also presented in Figure 3. It was obtained by presenting the same stimulus without any changes at −45° to 10 of 33 participants, providing a reference containing no ACC for comparisons with other location change evoked ACC waveforms.

**TABLE 3:** Mean and SD of N1′–P2′ amplitude and N1′ latency and P2′ latency in different conditions.

Angle (°)	Direction	Electrode site	N1′–P2′ amplitude (μV)	N1′ latency (ms)	P2′ latency (ms)
±2	Rightward	Cz	2.08 ± 0.57	708.80 ± 23.38	756.90 ± 24.92
		Fz	2.15 ± 0.54	706.80 ± 23.50	759.90 ± 24.77
	Leftward	Cz	2.50 ± 0.96	706.08 ± 15.40	758.54 ± 33.28
		Fz	2.57 ± 1.03	704.77 ± 15.46	759.62 ± 33.59
±5	Rightward	Cz	3.23±1.45	696.63±15.98	762.25±18.65
		Fz	3.23 ± 1.50	685.59 ± 29.91	761.78 ± 18.94
	Leftward	Cz	3.02 ± 1.03	695.69 ± 15.99	757.25 ± 21.58
		Fz	3.12 ± 1.17	692.53 ± 24.37	757.22 ± 22.20
±15	Rightward	Cz	4.77±1.84	682.73±14.12	750.39±14.51
		Fz	4.91 ± 1.62	682.88 ± 14.17	749.94 ± 14.70
	Leftward	Cz	4.37 ± 1.47	679.30 ± 15.71	749.18 ± 18.47
		Fz	4.50 ± 1.62	679.18 ± 19.18	748.18 ± 17.45
±45	Rightward	Cz	5.69±1.69	662.27±15.24	739.00±14.76
		Fz	5.84 ± 1.71	663.21 ± 15.84	739.67 ± 15.35
	Leftward	Cz	5.13 ± 1.94	660.67 ± 15.85	731.48 ± 15.06
		Fz	5.06 ± 2.18	662.88 ± 16.20	731.73 ± 15.50
*p*-Value	*p* (direction)		0.053	0.97	0.076
		*p* (electrode site)	0.761	0.38	0.936

*The mean and SD of the N1′–P2′ amplitude, N1′ latency, and P2′ latency are shown in Table 3. It can be observed that the N1′–P2′ amplitude tended to increase with the increase of test angle while the N1′ latency and P2′ latency tended to shorten. The N1′–P2′ amplitude at electrode site Fz was slightly larger than that of Cz, but there was no significant difference in the following statistics. There was no statistical difference in waveform latency or amplitude between different sound location change directions.*

### No Difference Between the Direction of Sound Location Change in Acoustic Change Complex Waveform

Figure 3A presents the averaged waveform obtained from Cz under four rightward positions while Figure 3B illustrates the waveforms evoked by leftward positions. These panels show that both rightward and leftward sound location changes elicit an ACC response. In addition, the statistical analysis revealed that the direction of sound location change had no statistically significant influence on the N1′–P2′ amplitude [*F*(1,25) = 3.82, *p* = 0.053 > 0.05, η = 0.03], N1′ latency [*F*(1,125) = 0.001, *p* = 0.97 > 0.05, η < 0.001], or P2′ latency [*F*(1,125) = 3.20, *p* = 0.076 > 0.05, η = 0.03].

### No Difference Between Fz and Cz in Acoustic Change Complex Recording

The ACC waveforms obtained from the Fz and Cz electrode positions presented a similar morphology, in terms of the amplitude and latencies of the P1–N1–P2 complex. The differences in the onset response at Fz and Cz were not statistically significant, as supported by the statistical analysis: N1′–P2′ amplitude [*F*(1,125) = 0.09, *p* = 0.761 > 0.05, η = 0.001], N1′ latency [*F*(1,125) = 0.78, *p* = 0.38 > 0.05, η = 0.006], and P2′ latency [*F*(1,125) = 0.006, *p* = 0.936 > 0.05, η < 0.001].

### Acoustic Change Complex Waveform Amplitude and Latency Were Related to Different Sound Location Changes

Since there was no difference between rightward and leftward sound location change, ACC at different angles were analyzed together. Considering the low ACC elicitation rate at ±2° (rightward 30.3% and leftward 39.4%) but high at ±5° and above (rightward and leftward both 96.97%), the ACC waveforms at ±45°, ±15°, and ±5° of 33 participants were included in data analysis. Table 4 characterizes the morphology changes of the ACC elicited at different angles.

**TABLE 4:** Comparisons of ACC between different test angles at Cz.

	N1′–P2′ amplitude (μV)			N1′ latency (ms)			P2′ latency (ms)		
Angle (°)	MD	SE	*p*	MD	SE	*p*	MD	SE	*p*
±5 vs. ±15	−1.49	0.16	<0.001***	11.59	2.31	<0.001***	10.27	1.86	<0.001***
±15 vs. ±45	−0.81	0.15	<0.001***	18.85	1.79	<0.001***	13.39	1.65	<0.001***
±5 vs. ±45	−2.30	0.18	<0.001***	30.44	2.47	<0.001***	23.66	2.09	<0.001***

The table showed the comparison results of N1′–P2′ amplitude, N1′ latency, and P2′ latency under different test angles (±5°, ±15°, and ±45°) in the analysis of variance. It can be seen that the N1′–P2′ amplitude tends to increase with the increase of test angle, and there was a significant statistical difference (p < 0.001) between different test angles. Meanwhile, N1′ latency and P2′ latency tend to shorten with the increase of test angle, and there was a significant statistical significance (p < 0.001) between different test angles. Mean difference and standard error are reflected in the table. MD, mean difference; SE, standard error; ***, p < 0.001.

**FIGURE 4:**
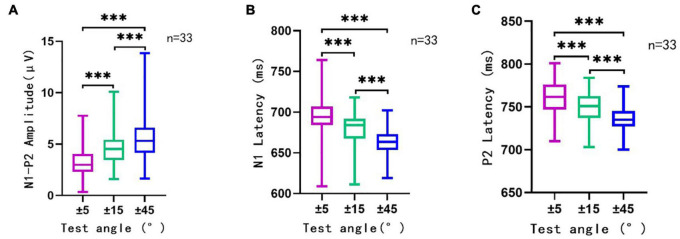
The N1′–P2′ amplitude **(A)**, N1′ latency **(B)**, and P2′ latency **(C)** in different conditions. This figure showed the N1′–P2′ amplitude, N1′ latency, and P2′ latency of ACC elicited by sound direction change at 500 ms (*n* = 33). Rightward and leftward location change-induced ACC at a certain angle were analyzed together as one group of data at that angle. The ordinate represented the amplitude **(A)** or latency **(B,C)** of ACC waveform. The abscissa represented different test angles. The purple line traced for a test angle change of ±5°, the green line traced for that of ±15°, and the blue line traced for ±45°. Mean, SD, and extreme value of the N1′–P2′ amplitude, N1′ latency, and P2′ latency are shown in this figure. It can be observed that with the increase in test angle, the N1′–P2′ amplitude showed a growing trend, and the latency of N1′ and P2′ experienced a decreasing trend. There was a significant statistical significance (*p* < 0.001) between different test angles in different conditions (±5° vs. ±15°, ±15° vs. ±45°, and ±5° vs. ±45°). ^***^, *p* < 0.001.

The ACC waveform amplitude increased with the angle of sound location change. Multi-factor repeated measurement analysis of variance was used to determine influences on ACC amplitude and latency from the direction of different sound location changes and electrode sites (Cz and Fz) with the degree of sound location change (±45°, ±15°, and ±5°). The N1′–P2′ amplitude showed a growing trend, and the latency of N1′ and P2′ experienced a decreasing trend (Figure 4) as the test angle increased. The degree of sound location change (±45°, ±15°, and ±5°) was the within-subjects variables, which had a statistically significant influence on the N1′–P2′ amplitude [*F*(1.91,238.1) = 97.172, *p* < 0.001, η = 0.44], N1′ latency [*F*(1.78,221.90) = 96.96, *p* < 0.001, η = 0.44], and P2′ latency [*F*(1.87,233.11) = 79.97, *p* < 0.001, η = 0.39].

### Minimum Audible Angle and Acoustic Change Complex

A total of 33 participants’ MAA on the horizontal plane was 2.09 ± 0.51°, where 97% of the participants could discriminate sounds coming from different locations at 2° in a two-alternative forced-choice task and only one participant’s MAA was 5°. Due to the size and accuracy of this study’s test system, 2° was the minimum angle that could be tested and measured. Other studies reported similar MAA values which ranged from 1° to 2° depending on the size and setting of the test system (Senn et al., 2005).

The ACC elicitation rate under each test condition is shown in Table 5. Results show that the change in horizontal sound location could successfully evoke an ACC. The magnitude of the ACC increased with the increase of sound location change. At ±45° and ±15° test angles, all 33 participants had clear ACC waveform with a 100% elicitation rate; at ±5° and 32 participants had clear waveform with a 97.0% elicitation rate. When the test angle was ±2°, approximately 40% of them generated a clear waveform.

**TABLE 5:** The number and proportion of participants with a clear waveform in each condition.

Angle (°)	Number of participants (elicitation rate)							
	Rightward location change				Leftward location change			
	Cz		Fz		Cz		Fz	
±2	10	(30.30%)	10	(30.30%)	13	(39.39%)	13	(39.39%)
±5	32	(96.97%)	32	(96.97%)	32	(96.97%)	32	(96.97%)
±15	33	(100%)	33	(100%)	33	(100%)	33	(100%)
±45	33	(100%)	33	(100%)	33	(100%)	33	(100%)

*This table shows the elicitation rate of ACC at different test angles (±2°, ±5°, ±15°, and ±45°). When the test angles were ±45° and ±15°, a clear ACC waveform can be elicited in all 33 participants. From ±5°, the elicitation rate began to decrease. With the decrease in the test angle, the elicitation rate of ACC decreased. The results were the same under different electrode sites (Cz and Fz). The results were similar (difference of three participants) under different sound directions (leftward and rightward).*

## Discussion

This study aimed to evaluate whether and how location change affected ACC and the relationship between MAA thresholds and objective ACC test to sound localization discrimination. Results demonstrated that horizontal sound location change can trigger ACC in young adults and most adults were able to elicit robust ACC at ±5° and above. N1′–P2′ amplitude increased and N1′ and P2′ latency decreased as the degree of change increased. There was no statistical difference in ACC waveform between rightward and leftward location change. The results of this study are discussed within relevant background literature, which is summarized in Table 6.

**TABLE 6:** Summary of the findings in other ACC studies.

Stimulus change	References	Subjects	Results
Location and frequency	Zhang et al., 2021	Normal hearing adults	Location change can elicit ACC and larger changes evoked a stronger activation. ACC mechanism involves memory-based acoustic comparison and involuntary attention switch.
Frequency, intensity and gap	He et al., 2012	Normal hearing adults	Electrophysiological thresholds were comparable to psychophysical thresholds for intensity discrimination but were higher than psychophysical thresholds for frequency or gap discrimination.
Speech syllables	Shetty and Puttabasappa, 2020	Hearing aid users	Latency of each component of ACC was significantly earlier and amplitude was higher in good hearing aid performers than poor hearing aid performers.

### Onset-Cortical Auditory-Evoked Potential

Cortical auditory evoked potential was evoked by the onset of the auditory stimulus as a sign of the stimulus being audible. The amplitude of CAEP at 45° was significantly higher than that of 5° and 15° which was most likely caused by the altered stimulus intensity in participants’ ears. The radial size of the test arc was relatively short in this study (1.2 m), making the sound transmission susceptible to the head size. The head radius would result in a slightly shorter distance between the speaker and the ipsilateral ear to it at 45° than 15° or 5°, causing a slightly louder stimulus to reach participants’ meatus. Although the intensity was calibrated at the center of the test arc before each test to ensure an unchanged presenting intensity, measurements *via* sound level meter confirmed that the intensity measured near the meatus of the ipsilateral ear to the presenting speaker at 45° was approximately 2 dB higher than that of 15° or 5°, which reached the just noticeable difference (Turner and Christopher, 1989; Martin and Boothroyd, 2000; Dimitrijevic et al., 2009). On the other hand, the time differences of sound reaching the ear from the speaker were negligible between different angles. Since the radius of the head was approximately 0.1 m in adults, the time difference caused by it was 0.29 ms (0.1 m/340 m/s). It was considerably smaller than normal hearing adults’ gap detection threshold, 4–5 ms (Samelli and Schochat, 2008), so it was unlikely to be recorded in participants. Furthermore, the latency of N1 and P2 remained unchanged regardless of the angle change which further proved the onset-CAEP amplitude increase was not caused by time difference or angle difference but by intensity difference alone. The onset-CAEP reflects that the detection of sound and its amplitude differences across different angles were caused by the different stimulus intensity at the nearest ear. To avoid the interference of intensity change, a sound localization system with a larger radius was needed to minimize the effect of head size in the actual stimulus spreading distance.

### The Acoustic Change Complex Evoked by Location Change

Acoustic change complex reflects the response to changes in physical characteristics of sounds on the auditory cortex level. The ACC can be triggered by changes in frequency, intensity, vowels, and other characteristics of sound, and reflects the sensitivity of the auditory cortex. This study demonstrated that the ACC could also be evoked by a horizontal sound location change, a result consistent with that of Zhang et al. (2021).

A sudden offset or an onset of a sound presented *via* speakers would normally result in a “click” noise but a programmed 1 ms overlap at the change was adopted in this study to provide a smooth sound transition which technically ensured that the ACC peak was not triggered by the click. Moreover, if the click still existed, this artifact would appear in all waveforms with a specific latency regardless of the different angle changes. However, it did not appear in the majority at 2° with an ACC elicitation rate of less than 40% and its latency increased as the location angle change became smaller. Therefore, it can be concluded that the ACC peak was not triggered by the click in this study.

Although the amplitude of the onset-CAEP was affected by the sound intensity at different angles, the ACC peak was still considered to be triggered by the location change rather than the intensity differences. Since the stimuli used in this study were symmetrically positioned, their acoustic features (including intensity) should be the same at ear level, except for the different locations. For example, when the stimulus moved from −45° to +45°, the overall intensity and arrival time were unchanged due to the symmetrical position of the loudspeaker, suggesting that ACC was elicited by the location change for each test condition. Thus, the amplitude of ACC was not caused by the monaural intensity change like onset-CAEP but mainly the location change. The latency of ACC followed the same principle that it increased as the angle change decreased. However, it could also be argued that lower stimulus levels would elicit a lower amplitude ACC; therefore, the ACC amplitude decreased as reported in this study as the angle difference reduction could be partially influenced by the lower levels presented in the narrower angle conditions. However, since the level differences between testing conditions were in the range of 1–2 dB, we expect such possible effect of level on the ACC morphology to be negligible (He et al., 2012).

Hence, this study ascertained that ACC can be triggered by the location change, and its interpretation should rely on both the presence of expected amplitude and latency and the expected changing trend as the change became more prominent.

Results indicated that the degree of sound location angle change had a significant effect on N1′–P2′ amplitude, N1′ latency, and P2′ latency of ACC. With an angle-change increase, N1′–P2′ amplitude showed an increasing trend, and N1′ and P2′ latency showed a shortening trend. This agreed with previous studies which used frequency or intensity as the stimulus for physical change (Martin and Boothroyd, 2000; He et al., 2012; Vonck et al., 2019). A similar response trend was also reported in other electrophysiological spatial studies where increased lateralization *via* inserting earphones (e.g., larger binaural cues) caused a larger EEG response (Ozmeral et al., 2019) or a stronger MEG attenuation (Salminen et al., 2010). And this common outcome was tested in free field conditions as well (Briley et al., 2013).

The emergence of ACC might indicate a combination of onset response and offset response when the sound’s physical characteristic changes, rather than an accidental change in the total number of synchronously excited neurons (Martin and Boothroyd, 2000). Magezi and Krumbholz (2010) and Salminen et al. (2010) hypothesized that the extraction of interaural time difference (ITD) in humans could be better explained by the opponent-channel model, where sound location is encoded in the firing rates of two opponent neural populations, which contrasts with the previously used Jeffress model of narrowly tuned neurons. It should also be noted that this opponent-channel model may not fully apply to the coding of interaural level difference (ILD), as there was little cortical structure overlap between these two cues (Ozmeral et al., 2019). Nevertheless, varying degrees of sound position change produces varying degrees of interaural signal differences, which in turn cause differences in the rate of neuronal firing of the corresponding nuclei and their conduction pathways (Grothe et al., 2010). ACC waveform differences induced from different angle-change reflect the amount of excitation–inhibition process of the auditory cortical neurons involved in sound localization, especially the degree of excitement for the difference in binaural signals, which is in line with the property of ACC that the waveform clarity increased as the change became more significant.

### Subjective and Objective Measurements of Sound Localization Discrimination

Acoustic change complex waveforms were evoked in all participants at ±45° and ±15° and the elicitation rate was 97.00% at ±5° but only 30.30–39.39% at ±2°. With the decrease of the test angle, the difficulty for participants to distinguish sound localization increased, and the elicitation rate of ACC decreased. This result indicated that most normal-hearing young people can distinguish sound localization changes at ±5° horizontal plane when recorded at the cortical level. Therefore, the ACC threshold of sound localization discrimination should be near ±5°.

Since the MAA threshold was 2.09 ± 0.51° in this study, we noted that sound localization discrimination recorded by the ACC test is higher relative to the behavioral threshold. Several studies have investigated the relationship between ACC as an objective electrophysiological measurement and psychophysical measurements, and a significant relationship has been found in the dimension of intensity change and frequency change where the ACC threshold was found to be higher than the behavioral threshold (Martin and Boothroyd, 2000; Won et al., 2011; He et al., 2012). For example, +2 dB or −3 dB changes in sound intensity can trigger ACC, while behavioral studies have shown that humans can detect sound intensity changes in 0.2–0.5 dB (Martin and Boothroyd, 2000). Mathew et al. (2016) also suggested that a stronger pitch change would elicit ACCs of larger amplitude. However, the relationship between ACC and MAA in terms of sound location change has not been discussed before. Auditory discrimination and change detection should share a similar cortical processing mechanism (Zhang et al., 2021), suggesting some similar characteristics, and also a possible link between ACC and behavioral measurement regarding sound location change.

### Effect of Location Change Direction on Acoustic Change Complex Waveform

This study has also demonstrated that the horizontal sound location change to the left or right has no significant effect on the waveform. This is consistent with the fact that ACC is associated with auditory *discrimination* rather than *identification* ability, meaning that participants do not need to identify the location change direction to notice that the location has changed at their cortical level. Some researchers suggested that the left deviation elicits more significant mismatch negativity (MMN) waveform than the right-sided deviation (Kaiser et al., 2000), while others believed that the amplitude of the MMN is not affected by the direction of motion (Altman et al., 2010; Peter et al., 2019). A larger test angle and larger sample size may be required in future experiments to clarify whether change direction affects ACC waveform, but it is possible to further shorten test time by using a specific location change direction.

### Effect of Electrodes on Acoustic Change Complex Waveform

Results also showed that the effect of electrode site (Cz and Fz) on the ACC waveform was not statistically significant. Since the long-latency auditory-evoked potential is most pronounced at the fronto-central electrodes, Cz and Fz, are often used as recording points. Recordings from Cz usually present large magnitude waveforms in both MMN and P300 tests (Peter et al., 2019), so this position can be used as a standardized recording site in ACC tests. As for the inverting electrode, either mastoid could be selected although a slightly larger CAEP might be recorded using the contralateral mastoid for people with amplification devices (Lightfoot, 2016). Common clinical tests should have the characteristics of simple and quick, but the preparation time for the EEG cap is long so it cannot fully meet the needs of clinical work. In this study, six points on the scalp were selected as recording points, and Ag/AgCl electrodes were directly connected to the scalp instead of wearing an EEG cap for acquisition. This method offered the possibility for daily clinical sound localization testing, especially for pediatrics.

## Conclusion

This study innovatively explored ACC features with different sound localization changes in a clinical sound field setting, providing insights into a more comprehensive sound localization test battery. This study demonstrated that changes in horizontal sound location trigger a reliable and reproducible ACC response and that the morphology of the ACC is sensitive to change the angle. The N1′–P2′ amplitude increases and the N1′ and P2′ latencies decrease as the degree of change increases. This study also compared the objective localization discrimination measurement (minimal angle change that elicited an ACC response) and the subjective localization discrimination measurement (MAA). Results show that the ACC threshold on most of the normal hearing participants was 5° and their MAA was approximately 2°, which is consistent with previous research showing that the objective threshold is slightly higher than the subjective threshold. The methodologies described in this study open new opportunities to develop a clinical test for objective sound-localization ability, which is especially appropriate for children and other non-collaborative individuals. The clinical translation of this methodology shall require further research efforts aimed at increasing the time efficiency of the proposed test paradigm, evaluating the sensitivity of the ACC to different hearing disorders, and exploring the sensitivity of the ACC to vertical changes of sound sources.

## Data Availability Statement

The raw data supporting the conclusions of this article will be made available by the authors, without undue reservation.

## Ethics Statement

The studies involving human participants were reviewed and approved by the Ethics Committee of Beijing Chaoyang Hospital. The patients/participants provided their written informed consent to participate in this study.

## Author Contributions

JZ and N-YW initiated the scientific question. MS, JV, and Q-JF provided the scientific advice about the electrophysiological test paradigm. Z-TF and J-XL designed and conducted the EEG recording test and performed the data analysis and interpretation with JV, under the supervision of N-YW and JZ. HL and L-YF conducted the participant preparation and facilitated ethical processing. XF and X-YG provided the facility support and sound field calibration. Z-HZ wrote the manuscript. X-LZ proofread the manuscript. All authors agreed to be accountable for the content of the work and approved the final version of the manuscript.

## Conflict of Interest

The authors declare that the research was conducted in the absence of any commercial or financial relationships that could be construed as a potential conflict of interest.

## Publisher’s Note

All claims expressed in this article are solely those of the authors and do not necessarily represent those of their affiliated organizations, or those of the publisher, the editors and the reviewers. Any product that may be evaluated in this article, or claim that may be made by its manufacturer, is not guaranteed or endorsed by the publisher.
